# Effect of different schedules of ten-valent pneumococcal conjugate vaccine on pneumococcal carriage in Vietnamese infants: results from a randomised controlled trial

**DOI:** 10.1016/j.lanwpc.2022.100651

**Published:** 2022-12-03

**Authors:** Heidi Smith-Vaughan, Beth Temple, Vo Thi Trang Dai, Pham Thi Hoan, Ho Nguyen Loc Thuy, Thanh V. Phan, Kathryn Bright, Nguyen Trong Toan, Doan Y. Uyen, Cattram Duong Nguyen, Jemima Beissbarth, Belinda Daniela Ortika, Monica Larissa Nation, Eileen Margaret Dunne, Jason Hinds, Jana Lai, Catherine Satzke, Tran Ngoc Huu, Kim Mulholland

**Affiliations:** a Menzies School of Health Research, Charles Darwin University, Darwin, NT, Australia; b Department of Infectious Disease Epidemiology, London School of Hygiene & Tropical Medicine, London, UK; c Infection and Immunity, Murdoch Children's Research Institute, Melbourne, VIC, Australia; d Department of Microbiology and Immunology, Pasteur Institute of Ho Chi Minh City, Ho Chi Minh City, Viet Nam; e Department of Disease Control and Prevention, Pasteur Institute of Ho Chi Minh City, Ho Chi Minh City, Viet Nam; f Department of Paediatrics, The University of Melbourne, Melbourne, VIC, Australia; g Institute for Infection and Immunity, St George's, University of London, London, UK; h BUGS Bioscience, London Bioscience Innovation Centre, London, UK; i Department of Microbiology and Immunology, The University of Melbourne at the Peter Doherty Institute for Infection and Immunity, Melbourne, VIC, Australia

**Keywords:** Pneumococcal conjugate vaccine, Pneumococcal carriage, Vaccine schedules

## Abstract

**Background:**

WHO recommends a three-dose infant pneumococcal conjugate vaccine (PCV) schedule administered as a two-dose primary series with booster (2 + 1) or a three-dose primary series (3 + 0). Data on carriage impacts of these and further reduced PCV schedules are needed to inform PCV strategies. Here we evaluate the efficacy against carriage of four different PCV10 schedules.

**Methods:**

Participants within an open-label, randomised controlled trial in Ho Chi Minh City, Vietnam, were allocated to receive PCV10 in a 3 + 1 (2,3,4,9 months, n = 152), 3 + 0 (2,3,4 months, n = 149), 2 + 1 (2,4,9.5 months, n = 250) or novel two-dose (2,6 months, n = 202) schedule, or no infant doses of PCV (two control groups, n = 197 and n = 199). Nasopharyngeal swabs collected between 2 and 24 months were analysed (blinded) for pneumococcal carriage and serotypes. Trial registration: ClinicalTrials.gov NCT01953510.

**Findings:**

Pneumococcal carriage prevalence was low (10.6–14.1% for vaccine-type (VT) at 12–24 months in unvaccinated controls). All four PCV10 schedules reduced VT carriage compared with controls (the 2 + 1 schedule at 12, 18, and 24 months; the 3 + 1 and two-dose schedules at 18 months; and the 3 + 0 schedule at 24 months), with maximum reductions of 40.1%–64.5%. There were no differences in VT carriage prevalence at 6 or 9 months comparing three-dose and two-dose primary series, and no differences at 12, 18, or 24 months when comparing schedules with and without a booster dose.

**Interpretation:**

In Vietnamese children with a relatively low pneumococcal carriage prevalence, 3 + 1, 2 + 1, 3 + 0 and two-dose PCV10 schedules were effective in reducing VT carriage. There were no discernible differences in the effect on carriage of the WHO-recommended 2 + 1 and 3 + 0 schedules during the first two years of life. Together with the previously reported immunogenicity data, this trial suggests that a range of PCV schedules are likely to generate significant direct and indirect protection.

**Funding:**

10.13039/501100000925NHMRC, 10.13039/100000865BMGF

**Research in context**

**Evidence before this study**

Pneumococcal conjugate vaccines (PCV) prevent pneumococcal disease both through direct protection of vaccinees and through indirect (herd) protection of the broader population by a reduction in pneumococcal carriage and transmission of vaccine serotypes. It is not usually possible to compare the efficacy of different PCV formulations or schedules on disease endpoints, so immunogenicity and carriage endpoints are used to estimate the comparative direct and indirect effects, respectively. The World Health Organization (WHO) currently recommends provision of PCV in either a 2 + 1 schedule (a two-dose primary series with a booster dose) or a 3 + 0 schedule (a three-dose primary series with no booster dose). There is also growing interest in cost-saving reduced-dose PCV schedules. We searched PubMed from database inception to 26 November 2021 using search terms including, but not limited to, “pneumococcal conjugate vaccine” OR “PHiD-CV” (PCV10) AND “carriage”. Few studies have evaluated the effect of different PCV schedules on carriage. At the time this trial was designed (2012) there were only two published studies (from Fiji and The Gambia) evaluating the effect of different PCV schedules on carriage, both of which compared three-dose and two-dose primary series using PCV7. A systematic review of PCV dosing studies published in 2014 forms the basis of the current WHO recommendations. That review identified only two head-to-head carriage studies of 3 + 0 and 2 + 1 schedules, one of which compared pre-booster data from a 3 + 1 schedule with post-booster data from a 2 + 1 schedule, and the other of which was preliminary unpublished data from the trial we report here. Since that review there has been one study comparing these schedules. A trial in South Africa found that carriage of vaccine serotypes tended to be lower in participants who received a PCV10 schedule with a booster dose (3 + 1 or 2 + 1) than one without a booster dose (3 + 0), albeit with widely overlapping confidence intervals.

**Added value of this study**

This study reports the final data from one of the two head-to-head carriage studies used to support the current WHO recommendation of 2 + 1 or 3 + 0 schedules for PCV. It also includes the manufacturer-recommended 3 + 1 schedule and a novel two-dose schedule at 2 and 6 months. Furthermore, this study includes an unvaccinated comparator group, allowing a fuller understanding of the relative impact of the different schedules. Given the importance of the herd protection effects, this study provides valuable data for decision-makers to support the choice of paediatric schedules for PCV.

**Implications of all the available evidence**

The results from this and previous studies show that 2 + 1 and 3 + 0 schedules have a similar effect on pneumococcal carriage and support current WHO recommendations. Both schedules reduce the carriage of vaccine serotypes during the second year of life, as do a 3 + 1 schedule and a two-dose schedule at 2 and 6 months. These results, coupled with the promising immunogenicity of the two-dose schedule from this trial, highlight the potential of reduced-dose schedules, which should be explored further. Data directly comparing different PCV schedules are crucial to show the comparative direct and indirect effects that can be expected, generating evidence to support decisions regarding the introduction and ongoing use of infant PCV both in Vietnam and elsewhere.

## Introduction

Pneumococcal conjugate vaccines (PCVs) are an important measure in preventing pneumococcal disease through direct protection as well as through indirect (herd) protection facilitated by reduced nasopharyngeal carriage of vaccine-types in a population.^1^^,^^2^ Infant PCV schedules still garner much attention as pneumococcal infections remain a major cause of morbidity and mortality in children under 5 years of age globally.^3^ In addition, young children are the main reservoir and key transmitters of this pathogen.^4^

Two PCVs are currently in widespread use globally; a 10-valent PCV (PCV10, Synflorix®, GSK Vaccines) protecting against serotypes 1, 4, 5, 6B, 7F, 9V, 14, 18C, 19F, and 23F, and a 13-valent PCV (PCV13, Prevenar®, Pfizer) containing the ten serotypes in PCV10 plus serotypes 3, 6A and 19A. A third PCV (Pneumosil®, Serum Institute of India), containing serotypes 1, 5, 6A, 6B, 7F, 9V, 14, 19A, 19F, and 23F received World Health Organization (WHO) pre-qualification in December 2019. Children in much of the world remain unvaccinated against pneumococcal disease due to the cost of national PCV programs. Recent WHO Position Papers on PCVs recommend a three-dose infant schedule administered either as a two-dose primary series followed by a booster (2 + 1) or as a three-dose primary series (3 + 0).^5^^,^^6^ The current recommendations are based on a 2017 systematic review,^7^ which identified several comparative immunogenicity trials for these schedules but only limited and inconclusive comparative carriage data. Two head-to-head carriage studies were identified: one compared post-booster data with a 2 + 1 schedule and pre-booster data with a 3 + 1 schedule,^8^ finding similar vaccine-type (VT) carriage rates in both groups each of which was reduced compared with controls; and the other was preliminary unpublished data from the trial we now report here. Since then one trial, from South Africa, has compared the effect of the two WHO-recommended schedules on carriage, finding similar VT carriage rates following a 3 + 1, 3 + 0, or 2 + 1 schedule of PCV10.^9^

An understanding of the effect of different PCV schedules on pneumococcal carriage is essential, as this is the mechanism through which herd protection is afforded, extending the benefits of vaccination beyond vaccinees to the broader population.^10^ Investigations of the timing and number of doses, accounting for local disease epidemiology and alignment with existing national immunisation programs, generate important data to inform the choice of paediatric schedules for PCVs. In 2020, the United Kingdom became the first country to implement a 1 + 1 PCV schedule. That decision was based on favourable post-booster immunogenicity compared with a 2 + 1 schedule^11^ and a belief that a 1 + 1 schedule is sufficient to maintain established herd protection. As interest in cost-saving reduced-dose PCV schedules continues to grow, data on the impacts of reduced schedules on pneumococcal carriage are needed.

In Vietnam, where pneumococcal conjugate vaccines are only available on the private market, pneumococcal disease remains a serious health issue. We undertook a randomised controlled trial of different PCV10 schedules in Ho Chi Minh City, Vietnam that included the manufacturer-recommended 3 + 1 schedule, the WHO-recommended 2 + 1 and 3 + 0 schedules, a novel two-dose schedule, and unvaccinated controls. Previously, we found all these schedules to be immunogenic.^12^ Here we report the carriage findings. The main objective was to assess the effect of the WHO-recommended 2 + 1 and 3 + 0 schedules and further reduced schedules on vaccine-type (VT) carriage to support decisions regarding infant PCV schedules.

## Methods

### Study design and participants

An open-label, randomised controlled trial was conducted in Ho Chi Minh City, Vietnam. A detailed protocol describing the trial aims, study design, study population, and sample size has been published previously.^13^ Infants were enrolled at two months of age and randomised to one of six vaccination schedules (Appendix Table S1): a 3 + 1 PCV10 schedule at 2, 3, 4, and 9 months of age (Group A), a 3 + 0 PCV10 schedule at 2, 3, and 4 months of age (Group B), a 2 + 1 PCV10 schedule at 2, 4, and 9.5 months of age (Group C), a two-dose PCV10 schedule at 2 and 6 months of age (Group D), a 2 + 1 PCV13 schedule at 2, 4, and 9.5 months of age (Group E) and a control group that received two doses of PCV10 (given at 18 and 24 months of age, Group F). Follow-up was initially scheduled until 18 months of age but later extended to 24 months. As the original control group (Group F) received PCV at 18 months, this necessitated recruitment of an additional group at 18 months of age to serve as unvaccinated controls between 18 and 24 months (Group G). Group G participants received a single dose of PCV10 at 24 months of age.

Here we describe the microbiological outcomes for the four different infant PCV10 schedules and unvaccinated controls (all groups except Group E). The comparative effect of 2 + 1 schedules of PCV10 and PCV13 on pneumococcal carriage were reported previously.^14^ Ethical approval was obtained from the Human Research Ethics Committee of the Northern Territory Department of Health and Menzies School of Health Research, Australia, and the Ministry of Health Ethics Committee, Vietnam. The trial is registered at ClinicalTrials.gov, number NCT01953510.

### Randomisation and masking

Participants were block randomised, stratified by district, to one of groups A to F (3:3:5:4:5:4) using a computer-generated list of randomisation numbers. Group G participants were recruited at 18 months of age from the study districts, concurrent with group A-F participants turning 18 months of age. All laboratory-based outcome assessors were masked to the group allocation. Additional details of the randomisation and masking were described previously.^13^

### Study procedures and laboratory analyses

Demographic data collected on forms were double-entered into an EpiData v3.1 database, with validation checks completed before upload into a Microsoft Access database. Laboratory data were entered into Microsoft Access (2–12 month timepoints) or Excel (18 and 24 month timepoints) databases.

Nasopharyngeal swabs were collected at 2, 6, 9, 12, 18, and 24 months of age, stored, and tested consistent with WHO guidelines.^15^ Samples collected at 2, 6, 9, and 12 months were cultured on Columbia Colistin Naladixic Acid Horse Blood agar plates, and presumptive *Streptococcus pneumoniae* identified based on colony morphology, α-haemolysis and susceptibility to optochin.^16^ Serotyping was conducted on isolates using latex agglutination and Quellung reaction.^17^^,^^18^ At 18 and 24 months samples were subject to quantitative real-time PCR (qPCR) targeting the autolysin (*lytA*) gene.^19^ Samples with presumptive pneumococci (*lytA* positive or equivocal) were cultured on selective agar before molecular serotyping by microarray (Senti-SP version 1.5, BUGS Bioscience).^20^ Pneumococci were designated as non-typeable if no serotype was identified using phenotypic testing, or if microarray identified a non-encapsulated lineage (NT1, NT2, NT3a, NT3b, NT4a, NT4b). Serotypes 15B and 15C were reported as 15B/C as these serotypes are known to interconvert,^21^ and ‘11F-like’ was reported as 11A.^22^ Serotype-specific density was derived by multiplying the overall pneumococcal density with the relative abundance of the serotype as determined by microarray.

### Outcomes

Vaccine-type (VT) carriage was defined as carriage of a serotype included in PCV10 (1, 4, 5, 6B, 7F, 9V, 14, 18C, 19F, and 23F). Non-VT carriage was defined as carriage of a serotype not in PCV10. Samples that contained both VT and non-VTs were considered positive for both vaccine-type and non-vaccine-type carriage. Carriage of serotypes 6A or 19A was reported both within non-VT carriage and as a separate category, as PCV10 may offer cross-protection against these serotypes.

### Statistical analyses

Analyses were conducted on the per-protocol population, in line with the primary immunological non-inferiority analysis from the trial, and in accordance with the statistical analysis plan. We determined the prevalence of any pneumococcal serotype carriage, VT carriage, non-VT carriage and serotype 6A/19A carriage at 2, 6, 9, 12, 18 and 24 months of age in each group. Prevalence was calculated as the number of carriers divided by the total number of participants for whom a microbiology result was available, expressed as a percentage with 95% confidence interval (CI). Each of the vaccine groups were compared with controls using prevalence ratios (PRs) and one-sided Fisher's Exact tests, expressed as percent reductions ([1-PR]×100) with 90% CIs representing vaccine efficacy against carriage. The control group used for comparison varied by timepoint based on vaccination status: Group F at 2–12 months, Groups F and G combined at 18 months, or Group G at 24 months. The sample size was based on the immunogenicity outcomes, but provided 64% power to detect a 40% reduction in VT carriage, assuming 24% VT carriage prevalence among controls. We also directly compared schedules with two-dose and three-dose primary series (Group C versus Groups A and B combined), and schedules with and without booster doses (Group A, or Groups A and C combined, versus Group B) using PRs with 95% CIs and two-sided Fisher's Exact tests. As a single measure of the effect of vaccination up to 18 months of age, we also determined the overall prevalence of carriage (with 95% CI) between 6 months of age (post-primary series) and 18 months of age (the time of first PCV dose in controls), with participants defined as carriers if they had a positive swab at any timepoint. Density was assessed at 18 and 24 months of age in pneumococcal carriers. Density data for pneumococcal carriers were log_10_-transformed and reported as log_10_ genome equivalents per ml (log_10_ GE/ml). As the transformed density data were not normally distributed, groups were compared using the non-parametric Mann–Whitney U test. Statistical analyses were conducted using Stata version 15.1 (StataCorp LLC).

### Role of the funding source

The funders of the study had no role in study design, data collection, data analysis, data interpretation, or writing of the report. The corresponding author had full access to all the data in the study and had final responsibility for the decision to submit for publication.

## Results

**Fig. 1:**
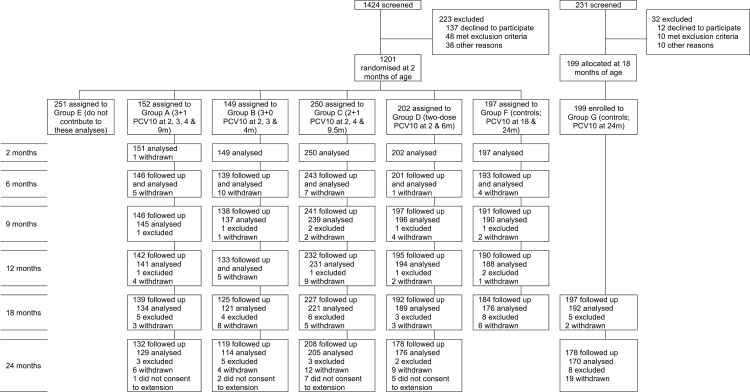
**CONSORT diagram**. 135 participants were withdrawn from the study for the following reasons: moved away and lost to follow-up (n = 80, 59%), refused a study procedure (n = 24, 18%), voluntary withdrawal (n = 19, 14%), and other (n = 12, 9%). 63 participants were excluded from analyses for the following reasons: no sample (either the participant missed the study visit or attended the visit but had no sample collected, n = 23), serotyping could not be conducted or a serotyping result could not be determined (n = 36), and protocol deviation (PCV was administered outside the trial [n = 2], sample was collected after administration of PCV [n = 1], and sample collected within 4 weeks of study PCV [n = 1]). Participants who “did not consent to extension” (n = 15) had their last sample collected at 18 months of age, as per the original study design. PCV10 = ten-valent pneumococcal conjugate vaccine.

Between Sept 30, 2013, and Jan 9, 2015, 1201 two-month-old infants were enrolled and randomised to Groups A-F (Fig. 1). The groups were balanced with respect to baseline characteristics, as previously reported.^13^ Between Apr 14, 2015, and May 12, 2016, 199 18-month-old PCV-naïve children were recruited to the additional control group (Group G). 1149 participants contribute data to this article (groups A [3 + 1 schedule, n = 152], B [3 + 0 schedule, n = 149], C [2 + 1 schedule, n = 250], D [two-dose schedule, n = 202], F [controls ≤18 months of age, n = 197], and G [controls ≥18 months of age, n = 199]). Baseline demographics were generally similar comparing participants recruited at 2 months and at 18 months of age (Appendix Table S2). The exceptions were sex and district, with fewer female participants and fewer participants from District 7 in Group G compared with the other groups. Participant characteristics at the time of each swab were similar across groups, with the exception of antibiotic use in the fortnight prior to the 2-month swab, which ranged from 1.3 to 6.6% (Table 1).

**Table 1:** Characteristics of participants analysed at each swab collection time point.

	3 + 1 schedule	3 + 0 schedule	2 + 1 schedule	Two-dose schedule	Controls^a^	p-value
**Age, months**						
2m	2.1 (1.9, 2.5)	2.1 (1.9, 2.4)	2.1 (1.9, 2.4)	2.1 (1.9, 2.4)	2.1 (1.9, 2.5)	>0.999
6m	6.1 (5.6, 7.9)	6.1 (5.8, 6.7)	6.1 (5.7, 6.9)	6.1 (5.6, 7.5)	6.1 (5.0, 6.8)	0.343
9m	9.1 (9.0, 11.0)	9.1 (8.9, 10.5)	9.1 (9.0, 10.1)	9.1 (9.0, 10.1)	9.1 (9.0, 11.2)	>0.999
12m	12.1 (11.3, 13.1)	12.1 (11.8, 12.6)	12.1 (12.0, 14.0)	12.1 (11.9, 13.8)	12.1 (12.0, 13.2)	>0.999
18m	18.1 (17.9, 19.6)	18.1 (17.8, 19.0)	18.1 (17.9, 20.9)	18.1 (17.9, 20.3)	18.2 (17.4, 20.3)	0.066
24m	24.1 (23.9, 26.6)	24.0 (23.8, 26.0)	24.1 (23.9, 25.9)	24.1 (23.9, 29.9)	24.1 (23.4, 26.9)	0.222
**Any current breastfeeding**						
2m	109/150^b^ (72.7%)	107/149 (71.8%)	195/250 (78.0%)	165/202 (81.7%)	140/196^b^ (71.4%)	0.074
6m	65/146 (44.5%)	70/139 (50.4%)	129/243 (53.1%)	107/201 (53.2%)	91/193 (47.2%)	0.386
9m	55/145 (37.9%)	63/137 (46.0%)	91/239 (38.1%)	81/196 (41.3%)	70/190 (36.8%)	0.466
12m	39/141 (27.7%)	48/133 (36.1%)	71/231 (30.7%)	65/194 (33.5%)	52/188 (27.7%)	0.406
18m	9/134 (6.7%)	13/121 (10.7%)	30/220^b^ (13.6%)	24/188^b^ (12.8%)	52/368 (14.1%)	0.228
24m	3/128^b^ (2.3%)	4/113^b^ (3.5%)	9/205 (4.4%)	9/175^b^ (5.1%)	10/170 (5.9%)	0.629
**Presence of URTI symptoms**						
2m	10/151 (6.6%)	15/149 (10.1%)	18/250 (7.2%)	14/202 (6.9%)	10/197 (5.1%)	0.510
6m	22/145^b^ (15.2%)	26/139 (18.7%)	43/243 (17.7%)	31/201 (15.4%)	27/193 (14.0%)	0.747
9m	25/145 (17.2%)	21/137 (15.3%)	38/239 (15.9%)	29/196 (14.8%)	28/190 (14.7%)	0.971
12m	28/141 (19.9%)	27/133 (20.3%)	50/231 (21.6%)	35/194 (18.0%)	34/188 (18.1%)	0.873
18m	23/134 (17.2%)	18/121 (14.9%)	23/220^b^ (10.5%)	35/188^b^ (18.6%)	59/368 (16.0%)	0.192
24m	24/128^b^ (18.8%)	21/113^b^ (18.6%)	31/205 (15.1%)	19/175^b^ (10.9%)	20/170 (11.8%)	0.176
**Antibiotic use in past fortnight**						
2m	10/151 (6.6%)	2/149 (1.3%)	6/250 (2.4%)	12/202 (5.9%)	4/197 (2.0%)	0.019
6m	9/146 (6.2%)	19/139 (13.7%)	22/243 (9.1%)	25/201 (12.4%)	17/193 (8.8%)	0.174
9m	19/145 (13.1%)	20/137 (14.6%)	36/239 (15.1%)	32/196 (16.3%)	26/190 (13.7%)	0.926
12m	12/141 (8.5%)	9/133 (6.8%)	25/231 (10.8%)	22/194 (11.3%)	22/188 (11.7%)	0.552
18m	14/134 (10.4%)	14/121 (11.6%)	28/220^b^ (12.7%)	21/188^b^ (11.2%)	59/368 (16.0%)	0.347
24m	15/128^b^ (11.7%)	11/113^b^ (9.7%)	18/205 (8.8%)	16/175^b^ (9.1%)	22/170 (12.9%)	0.672
**Current antibiotic use**						
2m	1/151 (0.7%)	0/149 (0.0%)	3/250 (1.2%)	3/202 (1.5%)	4/197 (2.0%)	0.467
6m	5/146 (3.4%)	11/139 (7.9%)	6/243 (2.5%)	9/201 (4.5%)	7/193 (3.6%)	0.131
9m	4/145 (2.8%)	8/137 (5.8%)	10/239 (4.2%)	15/196 (7.7%)	5/190 (2.6%)	0.112
12m	7/141 (5.0%)	8/133 (6.0%)	17/231 (7.4%)	9/194 (4.6%)	14/188 (7.4%)	0.689
18m	7/134 (5.2%)	7/121 (5.8%)	13/220^b^ (5.9%)	8/188^b^ (4.3%)	17/368 (4.6%)	0.928
24m	8/128^b^ (6.3%)	6/113^b^ (5.3%)	12/205 (5.9%)	6/175^b^ (3.4%)	10/170 (5.9%)	0.793

Data are median (range) or n/N (%). p-values based on quantile regression with bootstrapped standard errors (for comparisons of medians) or chi-squared test (for comparisons of proportions). URTI = upper respiratory tract infection (presence of runny nose and/or cough at the time of swab collection).

a Data for controls come from Group F (2–12 months), Groups F and G combined (18 months), or Group G (24 months).

b Data missing for one participant.

Of the 1149 participants, a total of 135 were withdrawn during the follow-up period, and a further 15 from groups A-D did not consent to the extended follow-up beyond 18 months (Fig. 1). In all, 97% of participants were followed up at 6 months (922 of 950 from groups A, B, C, D, and F), 96% at 9 months (913/950), 94% at 12 months (892/950), 93% at 18 months (1064 of 1149 from groups A, B, C, D, F, and G), and 87% at 24 months (815 of 937 from groups A, B, C, D, and G excluding those that did not consent to the extension). A total of 5532 swabs were collected, of which 5492 (99%) were included in the analyses. Of the 40 swabs not included, four were excluded due to protocol deviations and 36 because microbiology results were not available (pneumococcal carriage status could not be determined for nine, and serotyping could not be conducted for 27 swabs).

**Fig. 2:**
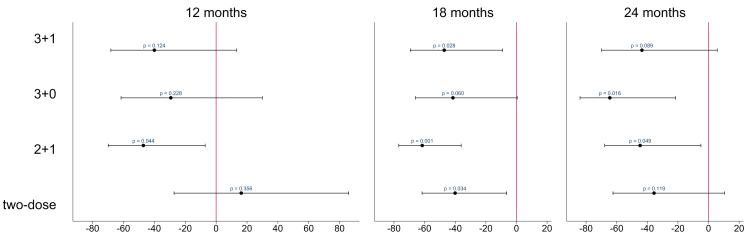
**Vaccine efficacy of PCV10 schedules on vaccine-type pneumococcal carriage**. Percent reduction (90% CI) in VT carriage of different PCV10 schedules at 12, 18 and 24 months compared with unvaccinated controls, calculated as ([1 – prevalence ratio] x100). P-values based on one-sided Fisher's exact test. Control group data came from Group F (2–12 months), Groups F and G combined (18 months), or Group G (24 months). PCV10 = ten-valent pneumococcal conjugate vaccine. CI = confidence interval. VT = vaccine-type.

We determined the vaccine efficacy of each of the different PCV10 schedules (3 + 1, 3 + 0, 2 + 1, and two-dose) on VT carriage at 12, 18, and 24 months of age, following completion of all vaccine doses (Fig. 2). All four schedules reduced VT carriage compared with unvaccinated controls, for whom VT carriage prevalence was 10.6% at 12 months, 14.1% at 18 months, and 12.4% at 24 months (Appendix Table S3). Across these three timepoints, point estimates ranged from a 40 to 47% reduction with a 3 + 1 schedule, a 29–64% reduction with a 3 + 0 schedule, a 45–62% reduction with a 2 + 1 schedule, and a 16% increase to a 40% reduction with a two-dose schedule (Fig. 2). Statistically significant reductions were observed at all three timepoints in the 2 + 1 group, at 18 months in the 3 + 1 and two-dose groups, and at 24 months in the 3 + 0 group, with non-significant reductions observed at most other timepoints (Fig. 2).

**Fig. 3:**
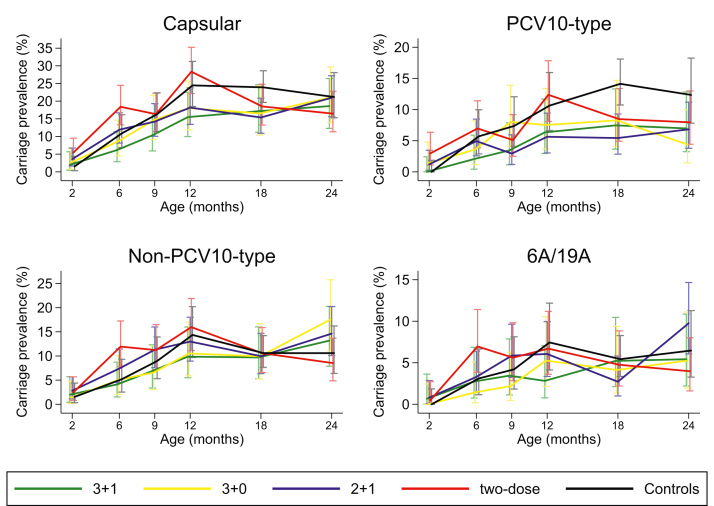
**Prevalence of pneumococcal carriage over time**. Carriage prevalence (95% CI) of any pneumococcal serotype, VT serotypes, non-VT serotypes, and serotype 6A or 19A over time for children receiving one of four PCV10 schedules or no PCV. Control group data came from Group F (2–12 months), Groups F and G combined (18 months), or Group G (24 months). CI = confidence interval. VT = vaccine-type. PCV = pneumococcal conjugate vaccine. PCV10 = ten-valent PCV.

We examined the patterns of pneumococcal carriage prevalence over time (Fig. 3). Across all groups, carriage of any pneumococcal serotype was low at 2 months of age, ranging from 1.5 to 5.4%, and peaked at 12 months of age, ranging from 15.5 to 28.4% (Appendix Table S3). The prevalence of VT carriage decreased between 6 and 9 months of age in both two-dose primary series groups (doses at 2 and 4 months of age in the 2 + 1 group or at 2 and 6 months of age in the two-dose group), a pattern that was not seen in the three-dose primary series or control groups (Fig. 3). VT carriage prevalence remained largely constant within each group between 12 and 24 months of age, although a decrease was observed in the two-dose group between 12 and 18 months, and in the 3 + 0 group between 18 and 24 months. No clear patterns emerged in the prevalence of non-VT or serotype 6A/19A carriage over time, although an increase in non-VT carriage was observed between 18 and 24 months of age among three of the vaccinated groups (3 + 1, 3 + 0 and 2 + 1). The most commonly carried serotypes were 6A, 6B, 23F, 19F, and 19A, which together accounted for 64% of pneumococci identified across all groups (Appendix Table S4). Serotype 3 was rarely detected throughout the study. Density was assessed at 18 and 24 months among children who carried pneumococci. There were no differences in VT, non-VT, or serotype 6A/19A density between any of the vaccinated groups and controls at 18 or 24 months (Appendix Fig. S1). Similar pneumococcal carriage density was observed at these two timepoints, with a median carriage density across all groups of 5.2 log_10_ GE/ml (IQR 4.5–5.8) at 18 months and 5.4 log_10_ GE/ml (4.6–6.1) at 24 months.

**Fig. 4:**
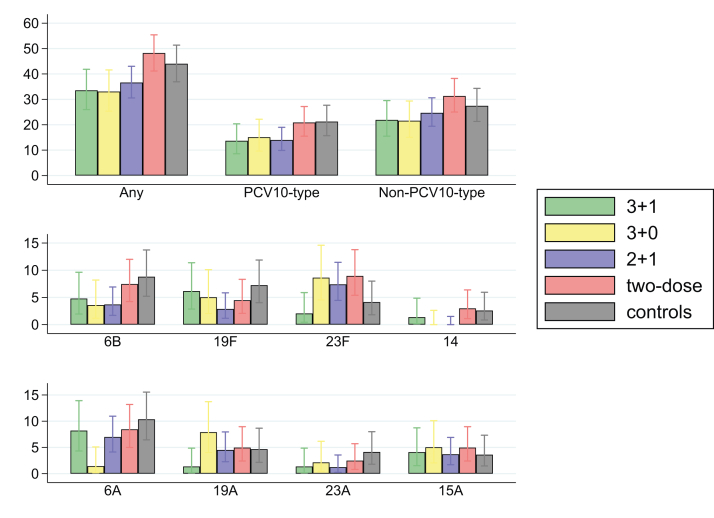
**Overall prevalence of pneumococcal carriage at any time between 6 and 18 months of age among the different PCV10 schedules**. Carriage prevalence (95% CI) of any pneumococcal serotype, VT serotypes, non-VT serotypes, and the eight most commonly carried serotypes at any time between 6 and 18 months of age. Control group data restricted to Group F. PCV10 = ten-valent pneumococcal conjugate vaccine. CI = confidence interval. VT = vaccine-type.

We also looked at the overall prevalence of carriage at any time between 6 and 18 months of age, of any pneumococcal serotype, any VT serotype, any non-VT serotype, and the most commonly carried four PCV10 serotypes and four non-PCV10 serotypes (Fig. 4). The overall prevalence of VT carriage was 13.7% (95% CI 8.6–20.4) in the 3 + 1 group, 15.1 (9.6–22.2) in the 3 + 0 group, 14.0 (9.9–19.0) in the 2 + 1 group, 20.9 (15.5–27.2) in the two-dose group, and 21.2 (15.7–27.7) in the control group. These represent reductions in the overall prevalence of VT carriage compared with unvaccinated controls of 36% (90% CI 3–57, p = 0.049) in the 3 + 1 group, 29% (−6 to 52, p = 0.101) in the 3 + 0 group, 34% (7–53, p = 0.031) in the 2 + 1 group. The overall prevalence of carriage of any pneumococcal serotype was 24% (90% CI 4–40, p = 0.032) and 25% (5–41, p = 0.028) lower in the 3 + 1 and 3 + 0 groups than in controls, respectively, with no differences between groups in the overall prevalence of non-VT carriage. The overall prevalence of serotype-specific carriage varied between serotypes and between groups, with values too low to draw conclusions.

To determine whether the number of primary series doses affects pneumococcal carriage during the interval between the primary series and the booster doses, we compared VT carriage prevalence between participants who received a three-dose (Groups A and B combined) or a two-dose (Group C) primary series of PCV10. There were no differences in the VT carriage prevalence between these groups at either 6 or 9 months of age (Appendix Table S5).

To determine whether the inclusion of a booster dose affects pneumococcal carriage up to two years of age, we compared VT carriage prevalence between participants who received a booster dose or no booster dose of PCV10. There were no differences in the VT carriage prevalence between those who received a 3 + 1 or 3 + 0 schedule (Group A vs Group B) at 12, 18, or 24 months of age (Appendix Table S6). In response to the lower-than-expected carriage prevalence observed across all groups we repeated the analysis comparing those who received either a 3 + 1 or a 2 + 1 schedule (Groups A and C combined) with those who received a 3 + 0 schedule (Group B), yielding the same results (Appendix Table S4). There were also no differences in carriage density (pneumococcal, VT, non-VT, or serotype 6A/19A) between groups that received a booster (3 + 1 or 3 + 1/2 + 1) compared with no booster (3 + 0) at either 18 or 24 months of age (Appendix Fig. S1).

## Discussion

Understanding the effect of alternative PCV schedules on carriage is an important factor for optimising paediatric immunisation programs. Such data are also essential for countries introducing PCVs, such as Vietnam where PCVs are not yet included in the national immunisation program. Here we provide data on the effect of PCV10 schedules with varied timing and number of doses on pneumococcal carriage to support PCV schedule design. This includes the final data from one of the two comparative trials that form the basis for the current WHO position on the comparative effect of 2 + 1 and 3 + 0 schedules on pneumococcal carriage, and the first evaluation of a two-dose schedule with a four-month interval.

We found that all four PCV10 schedules (3 + 1, 3 + 0, 2 + 1, two-dose) reduced carriage of PCV10 serotypes compared with an unvaccinated comparator group, suggesting that they are all likely to generate indirect herd protection effects. The 2 + 1 schedule, which had the largest number enrolled, reduced VT carriage at 12, 18 and 24 months of age. The other three schedules each reduced VT carriage at either 18 or 24 months of age, with trends towards reduced carriage at the other timepoints. These data complement our previously reported findings that all these schedules were immunogenic and likely to offer direct protection to vaccinees.^12^ We observed some increase in non-VT carriage between 18 and 24 months of age in three of the vaccinated groups (3 + 1, 3 + 0, 2 + 1) that was not seen in controls. It is not possible to determine if this trend would continue, but this observation highlights the importance of surveillance to monitor serotype replacement following PCV introduction.

Similar carriage prevalences were observed in the 2 + 1 and 3 + 0 groups, both post-primary series and post-booster. These findings are consistent with a trial in Finland^8^ that reported carriage rates post-booster with a 2 + 1 schedule and pre-booster with a 3 + 1 schedule (effectively 3 + 0 data, albeit from children 3 months younger than the 2 + 1 comparator group). In the Finnish trial, VT carriage rates were similar in both groups and were both reduced compared with unvaccinated controls. Similarly, a South African trial of PCV10 given in 3 + 1, 3 + 0 and 2 + 1 infant schedules identified no difference in VT carriage up to 24–27 months of age.^9^ A review of single arm and non-randomised vaccine effectiveness studies also support our findings^7^; both 2 + 1 and 3 + 0 schedules reduce VT carriage compared with unvaccinated controls, albeit with wide-ranging vaccine effectiveness estimates (19–88% for 2 + 1 schedules and 6–84% for 3 + 0 schedules). Meta-estimates of 41% (95% CI 28–59%) for the 2 + 1 studies and 24% (17–35%) for the 3 + 0 studies are suggestive of a greater effectiveness with 2 + 1 schedules, although these differences were not statistically significant. The same review also evaluated post-introduction impact studies, finding that neither schedule performed consistently better.^7^

Our study found no evidence for a difference in VT carriage in three-dose versus two-dose primary series of PCV10, either two or five months post-primary series. This is consistent with the Finnish study,^8^ which reported no difference in VT carriage either one or six months following a three-dose or two-dose primary series of PCV10. Previous cumulative systematic reviews on the comparative effect of three- and two-dose primary series on carriage from 2011,^23^ 2014,^24^ to 2016^25^ across studies from Fiji (PCV7), The Gambia (PCV7), Israel (PCV7) and South Africa (PCV10), all show a trend towards lower carriage following a three-dose primary series, although the only statistically significant difference was found at a single timepoint in the study from Fiji. The 2016 review included a meta-analysis of results four to seven months post-primary series from four studies, with a pooled relative risk of 0.81 (95% CI 0.64–1.02) for three-doses versus two-doses.^25^

We also examined a novel two-dose schedule with a four-month gap between doses. Our immunogenicity data from this trial^12^ showed that two doses with a four-month gap produced higher antibody levels than two doses with a two-month gap, but this was not reflected by lower carriage prevalence. Additionally, we showed that schedules containing a booster dose provided higher levels of antibodies up to age 18 months than those without a booster dose; however, there was no difference in VT carriage amongst participants who received a booster (3 + 1 or 3 + 1/2 + 1) versus a non-booster (3 + 0) series up to 24 months of age.

We did not identify any differences in pneumococcal carriage density between vaccinated groups and unvaccinated controls. Few data on density are available from randomised controlled trials; however our results are consistent with data from Fiji showing no effect of PCV on overall pneumococcal density in trial participants,^26^ and a study from Israel that found no difference in density (using semi-quantitative methods) of the six additional serotypes contained in PCV13 when comparing children who received PCV7 versus PCV13.^27^

The most commonly carried serotypes identified in this study were PCV10 serotypes 6B, 19F, and 23F, along with serotypes 6A and 19A. There are few other data on carriage or invasive pneumococcal disease (IPD) serotypes in Vietnam, but these results are consistent with a carriage study from 2008 to 2009 in Nha Trang^28^ and with IPD surveillance data from 2012 to 2016 in central and southern Vietnam,^29^ both of which found that serogroup 6 and serotypes 19F and 23F predominate.

This trial provided an opportunity to evaluate the impact of different schedules of PCV10 on pneumococcal carriage using an unvaccinated comparator group. A further strength was the follow-up to 24 months of age which enabled assessment of the effects of PCV schedules beyond infancy. A limitation was the lower than anticipated pneumococcal carriage prevalence, meaning that non-significant differences seen between groups may be due to a lack of power to detect these differences. This also meant that it was not possible to identify serotype-specific trends, and that results may not be generalisable to populations with higher carriage rates. A different control group was used at later timepoints, but few differences in characteristics between groups support this approach. Molecular microbiological methods were used only for the 18 and 24 month timepoints due to practical considerations, but this does not affect our conclusions as consistent methods were used across groups at any given timepoint.

In conclusion, we have shown that 3 + 1, 2 + 1, 3 + 0, and two-dose PCV10 schedules were effective in reducing VT carriage during the second year of life when compared with an unvaccinated comparator group. No difference was found between three-dose and two-dose primary series or between booster and non-booster schedules. These data support the use of either WHO-recommended 3 + 0 or 2 + 1 PCV schedule as well as the potential value of exploring further reduced schedules. Together with the immunogenicity data, this trial suggests that a range of PCV schedules are likely to generate significant direct and indirect protection. This is valuable information for those countries considering changes to PCV regimens or introduction of PCVs. More broadly, the findings from this trial suggest that a flexible approach to the timing of vaccine doses in national immunisation programmes could be an effective way to maximise protection at the population level.

## Contributors

BT and HSV co-wrote the manuscript with input from CS and KM. BT performed the statistical analyses with input from MN. HSV and CS oversaw the microbiology with JB, EMD, JH and BO. VTTD managed and performed laboratory testing at the Pasteur Institute laboratory with PTH, JL, TVP, and HNLT. CDN advised on the statistical plan and analyses. BT, MLN, JB and BO verified the underlying data. KB, NTT, and DYU were involved in the design, establishment, day-to-day management, and implementation of the trial. TNH was the site principal investigator, was involved in the design and establishment of the trial and had overall responsibility of its conduct in Vietnam. KM conceived the study, provided oversight for the conduct of the trial and the data analysis, and had overall responsibility for all aspects of the trial as the principal investigator. All authors contributed to refinement of and approved the submitted manuscript.

## Data sharing statement

The study protocol and informed consent form have been published previously and are freely available. Data will be made publicly available in accordance with the rules set out by the Bill & Melinda Gates Foundation and in line with human ethics approvals.

## Declaration of interests

The authors declare the following financial interests/personal relationships, which may be considered as potential competing interests: All authors except JB and JH received salary support from 10.13039/501100000925National Health and Medical Research Council of Australia (NHMRC) and/or 10.13039/100000865Bill & Melinda Gates Foundation grants. PCV10 vaccine doses were donated by GlaxoSmithKline Biologicals SA. KM, CS, and CN are investigators on a clinical research collaboration with Pfizer on PCV vaccination in Mongolia and are investigators on a Merck Investigator Studies Program grant funded by MSD on pneumococcal serotype epidemiology in children. JB provided a report on pneumococcal carriage in Northern Australia to MSD Australia. EMD is currently employed by Pfizer. JH receives project grant funding from 10.13039/100004319Pfizer and is co-founder and board member of BUGS Bioscience Ltd. We declare no other competing interests.

## Appendix A Supplementary data

Appendix Fig. S1 and Tables S1–S6

Caption for supplementary material

Temple-2018-Evaluation-of-different-infant-vacc
