# Supplementary material for: Effect of different schedules of ten-valent pneumococcal conjugate vaccine on pneumococcal carriage in Vietnamese infants: results from a randomised controlled trial
Source: Lancet Reg Health West Pac. 2022 Dec 3;32:100651. doi: 10.1016/j.lanwpc.2022.100651 (PMC9918756; doi:10.1016/j.lanwpc.2022.100651)
Supplement: Appendix Fig. S1 and Tables S1–S6 [file mmc1.docx]

**APPENDIX**

**Effect of different schedules of ten-valent pneumococcal conjugate vaccine on pneumococcal carriage in Vietnamese infants: results from a randomised controlled trial**

**TABLE OF CONTENTS**

[Appendix Table S1: Vaccination schedules and nasopharyngeal swabs in the Vietnam Pneumococcal Project 2](#_Toc114054675)

[Appendix Table S2: Participant demographics at the time of enrolment 3](#_Toc114054676)

[Appendix Table S3: Pneumococcal carriage prevalence by time point and vaccination group, % (95% CI) 4](#_Toc114054677)

[Appendix Table S4: Serotype-specific carriage prevalence over time 5](#_Toc114054678)

[Appendix Figure S1: Pneumococcal density at A) 18 months of age, and B) 24 months of age. 7](#_Toc114054679)

[Appendix Table S5: Comparative effect of a three-dose or two-dose primary series on VT carriage during the interval between the primary series and the booster doses 8](#_Toc114054680)

[Appendix Table S6: Effect of a booster-dose on VT carriage at 12, 18, and 24 months of age 8](#_Toc114054681)

# **Appendix Table S1: Vaccination schedules and nasopharyngeal swabs in the Vietnam Pneumococcal Project**

| **Time point** |  |  | **2m** | **3m** | **4m** |  | **6m** |  | **9m*** |  | **12m** |  | **18m** |  | **24m** |
| --- | --- | --- | --- | --- | --- | --- | --- | --- | --- | --- | --- | --- | --- | --- | --- |
| **NP swabs** |  |  | X |  |  |  | X |  | X |  | X |  | X |  | X |
| **PCV doses** |  |  |  |  |  |  |  |  |  |  |  |  |  |  |  |
| **Group** | **Schedule** | **Vaccine** |  |  |  |  |  |  |  |  |  |  |  |  |  |
| **A** | 3+1 | PCV10 | X | X | X |  |  |  | X |  |  |  |  |  |  |
| **B** | 3+0 | PCV10 | X | X | X |  |  |  |  |  |  |  |  |  |  |
| **C** | 2+1 | PCV10 | X |  | X |  |  |  | X |  |  |  |  |  |  |
| **D** | Two-dose | PCV10 | X |  |  |  | X |  |  |  |  |  |  |  |  |
| **E** | 2+1 | PCV13 | X |  | X |  |  |  | X |  |  |  |  |  |  |
| **F** | Controls | PCV10 |  |  |  |  |  |  |  |  |  |  | X |  | X |
| **G** ^†^ | Controls | PCV10 |  |  |  |  |  |  |  |  |  |  |  |  | X |

PCV = pneumococcal conjugate vaccine. PCV10 = ten-valent PCV. PCV13 = 13-valent PCV. NP = nasopharyngeal. * Booster dose of PCV administered at 9 months of age in group A and at 9·5 months of age in groups C and E. † Group G recruited at 18 months of age.

# **Appendix Table S2: Participant demographics at the time of enrolment**

|  | **3+1 schedule**  (Group A, n=152) | **3+0 schedule**  (Group B, n=149) | **2+1 schedule**  (Group C, n=250) | **two-dose schedule**  (Group D, n=202) | **controls 2-18m**  (Group F, n=197) | **controls 18-24m**  (Group G, n=199) | p-value* |
| --- | --- | --- | --- | --- | --- | --- | --- |
| Age, m [median (range)] | 2·1 (1·9, 2·5) | 2·1 (1·9, 2·4) | 2·1 (1·9, 2·4) | 2·1 (1·9, 2·4) | 2·1 (1·9, 2·5) | 18·3 (17·4, 20·3) | NA |
| Sex |  |  |  |  |  |  | 0·044 |
| Male | 66 (43·4%) | 73 (49·0%) | 135 (54·0%) | 91 (45·0%) | 100 (50·8%) | 113 (56·8%) |  |
| Female | 86 (56·6%) | 76 (51·0%) | 115 (46·0%) | 111 (55·0%) | 97 (49·2%) | 86 (43·2%) |  |
| District |  |  |  |  |  |  | 0·019 |
| 4 | 68 (44·7%) | 67 (45·0%) | 112 (44·8%) | 90 (44·6%) | 87 (44·2%) | 107 (53·8%) |  |
| 7 | 84 (55·3%) | 82 (55·0%) | 138 (55·2%) | 112 (55·4%) | 110 (55·8%) | 92 (46·2%) |  |
| Birthweight , g† [mean (sd)] | 3234 (424) | 3212 (349) | 3228 (370) | 3234 (410) | 3208 (395) | 3264 (423) | 0·198 |
| Place of delivery† |  |  |  |  |  |  | 0·605 |
| Hospital | 111 (73·0%) | 114 (76·5%) | 196 (78·7%) | 147 (72·8%) | 144 (73·1%) | 152 (76·8%) |  |
| Other | 41 (27·0%) | 35 (23·5%) | 53 (21·3%) | 55 (27·2%) | 53 (26·9%) | 46 (23·2%) |  |
| Type of delivery† |  |  |  |  |  |  | 0·088 |
| Vaginal | 89 (58·9%) | 85 (57·8%) | 160 (65·0%) | 130 (64·4%) | 121 (61·7%) | 104 (53·1%) |  |
| Elective caesarean | 30 (19·9%) | 30 (20·4%) | 43 (17·5%) | 36 (17·8%) | 34 (17·3%) | 44 (22·4%) |  |
| Emergency caesarean | 27 (17·9%) | 30 (20·4%) | 40 (16·3%) | 34 (16·8%) | 41 (20·9%) | 43 (21·9%) |  |
| Other/unknown | 5 (3·3%) | 2 (1·4%) | 3 (1·2%) | 2 (1·0%) | 0 (0·0%) | 5 (2·6%) |  |
| Cigarette smoker in house† |  |  |  |  |  |  | 0·951 |
| No | 57 (37·5%) | 52 (34·9%) | 81 (32·5%) | 74 (36·6%) | 72 (36·5%) | 70 (35·2%) |  |
| Yes | 95 (62·5%) | 97 (65·1%) | 168 (67·5%) | 128 (63·4%) | 125 (63·5%) | 129 (64·8%) |  |

Data are n (%) unless specified. * p-values compare combined data from Groups A, B, C, D, and F, with data from Group G, and are based on chi-squared test (for comparisons of proportions), ANOVA (for comparisons of means), or quantile regression with bootstrapped standard errors (for comparisons of medians). NA = not applicable, as participants were intentionally recruited at different ages. † Birthweight data missing for 11 participants (Group B=1, C=3, D=3, F=1, G=3); Place of delivery data missing for 2 participants (C=1, G=1); Type of delivery data missing for 11 participants (A=1, B=2, C=4, F=1, G=3); Cigarette smoker data missing for 1 participant (C=1).

# **Appendix Table S3: Pneumococcal carriage prevalence by time point and vaccination group, % (95% CI)**

|  | **3+1 schedule**  **(Group A)** | **3+0 schedule**  **(Group B)** | **2+1 schedule**  **(Group C)** | **Two-dose schedule**  **(Group D)** | **Controls*** |
| --- | --- | --- | --- | --- | --- |
| **Any pneumococcal serotype carriage** |  |  |  |  |  |
| 2m | 2·0 (0·4-5·7) | 2·7 (0·7-6·7) | 3·6 (1·7-6·7) | 5·4 (2·7-9·5) | 1·5 (0·3-4·4) |
| 6m | 6·2 (2·9-11·4) | 8·6 (4·5-14·6) | 11·9 (8·1-16·7) | 18·4 (13·3-24·5) | 10·9 (6·9-16·2) |
| 9m | 10·3 (5·9-16·5) | 14·6 (9·2-21·6) | 14·2 (10·1-19·3) | 16·3 (11·4-22·3) | 16·3 (11·4-22·4) |
| 12m | 15.6 (10.0-22.7) | 18·0 (11·9-25·6) | 18·2 (13·4-23·8) | 28·4 (22·1-35·2) | 24·5 (18·5-31·3) |
| 18m | 17·2 (11·2-24·6) | 16·5 (10·4-24·4) | 15·4 (10·9-20·8) | 18·5 (13·3-24·8) | 23·9 (19·6-28·6) |
| 24m | 18·6 (12·3-26·4) | 21·1 (14·0-29·7) | 21·0 (15·6-27·2) | 16·5 (11·3-22·8) | 21·2 (15·3-28·1) |
| **VT carriage** |  |  |  |  |  |
| 2m | 0·0 (0·0-2·4) | 1·3 (0·2-4·8) | 1·2 (0·2-3·5) | 3·0 (1·1-6·4) | 0·0 (0·0-1·9) |
| 6m | 2·1 (0·4-5·9) | 3·6 (1·2-8·2) | 4·9 (2·6-8·5) | 7·0 (3·9-11·4) | 5·7 (2·9-10·0) |
| 9m | 3·4 (1·1-7·9) | 8·0 (4·1-13·9) | 2·9 (1·2-5·9) | 5·1 (2·5-9·2) | 7·4 (4·1-12·1) |
| 12m | 6.4 (3.0-11.8) | 7·5 (3·7-13·4) | 5·6 (3·0-9·4) | 12·4 (8·1-17·8) | 10·6 (6·6-16·0) |
| 18m | 7·5 (3·6-13·3) | 8·3 (4·0-14·7) | 5·4 (2·8-9·3) | 8·5 (4·9-13·4) | 14·1 (10·7-18·1) |
| 24m | 7·0 (3·2-12·8) | 4·4 (1·4-9·9) | 6·8 (3·8-11·2) | 8·0 (4·4-13·0) | 12·4 (7·8-18·3) |
| **Non-VT carriage** |  |  |  |  |  |
| 2m | 2·0 (0·4-5·7) | 1·3 (0·2-4·8) | 2·8 (1·1-5·7) | 2·5 (0·8-5·7) | 1·5 (0·3-4·4) |
| 6m | 4·1 (1·5-8·7) | 5·0 (2·0-10·1) | 7·4 (4·4-11·5) | 11·9 (7·8-17·2) | 5·2 (2·5-9·3) |
| 9m | 6·9 (3·4-12·3) | 6·6 (3·0-12·1) | 11·3 (7·6-16·0) | 11·2 (7·2-16·5) | 8·9 (5·3-13·9) |
| 12m | 9·9 (5·5-16·1) | 10·5 (5·9-17·0) | 13·0 (8·9-18·0) | 16·0 (11·1-21·9) | 14·4 (9·7-20·2) |
| 18m | 9·7 (5·3-16·0) | 9·9 (5·2-16·7) | 10·0 (6·3-14·7) | 10·6 (6·6-15·9) | 10·6 (7·6-14·2) |
| 24m | 13·2 (7·9-20·3) | 17·5 (11·1-25·8) | 14·6 (10·1-20·2) | 8·5 (4·8-13·7) | 10·6 (6·4-16·2) |
| **Serotype 6A/19A carriage** |  |  |  |  |  |
| 2m | 0·7 (0·0-3·6) | 0·0 (0·0-2·4) | 0·8 (0·1-2·9) | 0·5 (0·0-2·7) | 0·0 (0·0-1·9) |
| 6m | 2·7 (0·8-6·9) | 1·4 (0·2-5·1) | 3·3 (1·4-6·4) | 7·0 (3·9-11·4) | 3·1 (1·1-6·6) |
| 9m | 3·4 (1·1-7·9) | 2·2 (0·5-6·3) | 5·9 (3·2-9·6) | 5·6 (2·8-9·8) | 4·2 (1·8-8·1) |
| 12m | 2·8 (0·8-7·1) | 5·3 (2·1-10·5) | 6·1 (3·4-10·0) | 6·7 (3·6-11·2) | 7·4 (4·1-12·2) |
| 18m | 5·2 (2·1-10·5) | 4·1 (1·4-9·4) | 2·7 (1·0-5·8) | 4·8 (2·2-8·8) | 5·4 (3·4-8·3) |
| 24m | 5·4 (2·2-10·9) | 5·3 (2·0-11·1) | 9·8 (6·1-14·7) | 4·0 (1·6-8·0) | 6·5 (3·3-11·3) |

* Control data sourced from Group F (2-12 months), Groups F and G combined (18 months), or Group G (24 months). Carriage determined by culture and latex agglutination/Quellung testing (2-12 months) and by DNA microarray (18-24 months). Samples that could not be serotyped are excluded. VT = vaccine type (PCV10 serotypes 1, 4, 5, 6B, 7F, 9V, 14, 18C, 19F, and 23F).

# **Appendix Table S4: Serotype-specific carriage prevalence over time**

**a) PCV10 and cross-reactive serotypes**

|  | **3+1 schedule**  **(Group A)** | **3+0 schedule**  **(Group B)** | | **2+1 schedule**  **(Group C)** | | **Two-dose schedule**  **(Group D)** | | **Controls*** |
| --- | --- | --- | --- | --- | --- | --- | --- | --- |
| **PCV10 serotypes** |  |  | |  | |  | |  |
| **6B** |  |  | |  | |  | |  |
| 2m | 0 | 0 | | 0.4 (1/250) | | 1.5 (3/202) | | 0 |
| 6m | 1.4 (2/146) | 1.4 (2/139) | | 1.2 (3/243) | | 2.5 (5/201) | | 2.6 (5/193) |
| 9m | 1.4 (2/145) | 2.2 (3/137) | | 1.3 (3/239) | | 3.6 (7/196) | | 3.2 (6/190) |
| 12m | 2.8 (4/141) | 0.8 (1/133) | | 2.6 (6/231) | | 5.2 (10/194) | | 4.3 (8/188) |
| 18m | 0.7 (1/134) | 1.7 (2/121) | | 0.9 (2/221) | | 1.6 (3/189) | | 4.1 (15/368) |
| 24m | 3.1 (4/129) | 1.8 (2/114) | | 1.5 (3/205) | | 1.1 (2/176) | | 5.3 (9/170) |
| **23F** |  |  | |  | |  | |  |
| 2m | 0 | 1.3 (2/149) | | 0.4 (1/250) | | 0.5 (1/202) | | 0 |
| 6m | 0 | 1.4 (2/139) | | 3.3 (8/243) | | 2.5 (5/201) | | 0.5 (1/193) |
| 9m | 0 | 4.4 (6/137) | | 1.3 (3/239) | | 0.5 (1/196) | | 0.5 (1/190) |
| 12m | 1.4 (2/141) | 3.0 (4/133) | | 2.2 (5/231) | | 4.6 (9/194) | | 1.6 (3/188) |
| 18m | 1.5 (2/134) | 3.3 (4/121) | | 2.7 (6/221) | | 4.8 (9/189) | | 4.3 (16/368) |
| 24m | 1.6 (2/129) | 0 | | 3.4 (7/205) | | 2.3 (4/176) | | 3.5 (6/170) |
| **19F** |  |  | |  | |  | |  |
| 2m | 0 | 0 | | 0 | | 1.0 (2/202) | | 0 |
| 6m | 0.7 (1/146) | 0.7 (1/139) | | 0.4 (1/243) | | 1.0 (2/201) | | 2.1 (4/193) |
| 9m | 2.1 (3/145) | 1.5 (2/137) | | 0.4 (1/239) | | 1.0 (2/196) | | 3.2 (6/190) |
| 12m | 2.1 (3/141) | 3.0 (4/133) | | 0.9 (2/231) | | 2.6 (5/194) | | 3.7 (7/188) |
| 18m | 3.7 (5/134) | 3.3 (4/121) | | 1.8 (4/221) | | 0.5 (1/189) | | 4.6 (17/368) |
| 24m | 1.6 (2/129) | 2.6 (3/114) | | 1.5 (3/205) | | 3.4 (6/176) | | 2.9 (5/170) |
| **14** |  |  | |  | |  | |  |
| 2m | 0 | 0 | | 0.4 (1/250) | | 0 | | 0 |
| 6m | 0 | 0 | | 0 | | 1.0 (2/201) | | 0.5 (1/193) |
| 9m | 0 | 0 | | 0 | | 0 | | 0 |
| 12m | 0 | 0 | | 0 | | 1.0 (2/194) | | 0.5 (1/188) |
| 18m | 1.5 (2/134) | 0 | | 0 | | 1.6 (3/189) | | 1.9 (7/368) |
| 24m | 0.8 (1/129) | 0 | | 0 | | 0 | | 2.4 (4/170) |
| **Other PCV10 serotypes** |  |  | |  | |  | |  |
| 2m | 0 | 0 | | 0 | | 0 | | 0 |
| 6m | 0 | 0 | | 0 | | 0 | | 0.5 (1/193) |
| 9m | 0 | 0 | | 0 | | 0 | | 0.5 (1/190) |
| 12m | 0 | 0.8 (1/133) | | 0 | | 0.5 (1/194) | | 0.5 (1/188) |
| 18m | 0 | 0 | | 0.5 (1/221) | | 0 | | 0 |
| 24m | 0 | 0.9 (1/114) | | 1.0 (2/205) | | 1.1 (2/176) | | 0 |
| **Cross-reactive serotypes** | | |  | |  | |  |  |
| **6A** |  |  | |  | |  | |  |
| 2m | 0.7 (1/151) | 0 | | 0 | | 0.5 (1/202) | | 0 |
| 6m | 2.1 (3/146) | 0.7 (1/139) | | 1.6 (4/243) | | 5.0 (10/201) | | 1.6 (3/193) |
| 9m | 3.4 (5/145) | 0 | | 3.3 (8/239) | | 4.1 (8/196) | | 3.2 (6/190) |
| 12m | 2.8 (4/141) | 0.8 (1/133) | | 3.0 (7/231) | | 3.6 (7/194) | | 5.9 (11/188) |
| 18m | 4.5 (6/134) | 0 | | 1.8 (4/221) | | 2.1 (4/189) | | 4.1 (15/368) |
| 24m | 3.9 (5/129) | 2.6 (3/114) | | 6.8 (14/205) | | 2.8 (5/176) | | 3.5 (6/170) |
| **19A** |  |  | |  | |  | |  |
| 2m | 0 | 0 | | 0.8 (2/250) | | 0 | | 0 |
| 6m | 0.7 (1/146) | 0.7 (1/139) | | 1.6 (4/243) | | 2.0 (4/201) | | 1.6 (3/193) |
| 9m | 0 | 2.2 (3/137) | | 2.5 (6/239) | | 1.5 (3/196) | | 1.1 (2/190) |
| 12m | 0 | 4.5 (6/133) | | 3.0 (7/231) | | 3.1 (6/194) | | 1.6 (3/188) |
| 18m | 0.7 (1/134) | 4.1 (5/121) | | 0.9 (2/221) | | 2.6 (5/189) | | 1.4 (5/368) |
| 24m | 1.6 (2/129) | 2.6 (3/114) | | 2.4 (5/205) | | 1.1 (2/176) | | 2.9 (5/170) |

Data are prevalence, expressed as a percentage, (n/N). * Control data sourced from Group F (2-12 months), Groups F and G combined (18 months), or Group G (24 months). Carriage determined by culture and latex agglutination/Quellung testing (2-12 months) and by DNA microarray (18-24 months). Samples that could not be serotyped are excluded. The **11 Other PCV10 serotypes** comprise: **5 x serotype 9V** (1 at 18m [C], 4 at 24m [B=1, C=1, D=2]), **3 x serotype 4** (1 at 9m [Controls], 1 at 12m [Controls], 1 at 24m [C]), **2 x serotype 18C** (1 at 6m [Controls], 1 at 12m [D]), and **1 x serotype 1** (12m [B]).

**b) PCV10 and cross-reactive serotypes**

|  | **3+1 schedule**  **(Group A)** | **3+0 schedule**  **(Group B)** | | **2+1 schedule**  **(Group C)** | | **Two-dose schedule**  **(Group D)** | | **Controls*** |
| --- | --- | --- | --- | --- | --- | --- | --- | --- |
| **Non-vaccine serotypes** |  |  | |  | |  | |  |
| **15A** |  |  | |  | |  | |  |
| 2m | 0 | 0 | | 0.8 (2/250) | | 0 | | 0.5 (1/197) |
| 6m | 0 | 0 | | 0.4 (1/243) | | 0.5 (1/201) | | 0.5 (1/193) |
| 9m | 0.7 (1/145) | 2.2 (3/137) | | 1.3 (3/239) | | 1.0 (2/196) | | 1.6 (3/190) |
| 12m | 2.8 (4/141) | 0.8 (1/133) | | 2.2 (5/231) | | 1.0 (2/194) | | 1.1 (2/188) |
| 18m | 2.2 (3/134) | 3.3 (4/121) | | 1.8 (4/221) | | 3.2 (6/189) | | 1.6 (6/368) |
| 24m | 0.8 (1/129) | 2.6 (3/114) | | 1.5 (3/205) | | 1.1 (2/176) | | 1.2 (2/170) |
| **23A** |  |  | |  | |  | |  |
| 2m | 0 | 0.7 (1/149) | | 0.4 (1/250) | | 0 | | 0 |
| 6m | 0 | 0.7 (1/139) | | 0.8 (2/243) | | 0.5 (1/201) | | 1.0 (2/193) |
| 9m | 0 | 1.5 (2/137) | | 0 | | 1.0 (2/196) | | 2.1 (4/190) |
| 12m | 0 | 0.8 (1/133) | | 0 | | 1.5 (3/194) | | 1.1 (2/188) |
| 18m | 1.5 (2/134) | 0 | | 0.9 (2/221) | | 1.1 (2/189) | | 1.1 (4/368) |
| 24m | 0 | 2.6 (3/114) | | 0.5 (1/205) | | 1.1 (2/176) | | 2.4 (4/170) |
| **11A** |  |  | |  | |  | |  |
| 2m | 1.3 (2/151) | 0 | | 0.8 (2/250) | | 0 | | 0 |
| 6m | 1.4 (2/146) | 0.7 (1/139) | | 1.2 (3/243) | | 0 | | 0 |
| 9m | 1.4 (2/145) | 0 | | 1.7 (4/239) | | 1.0 (2/196) | | 0 |
| 12m | 1.4 (2/141) | 2.3 (3/133) | | 1.7 (4/231) | | 0.5 (1/194) | | 0.5 (1/188) |
| 18m | 0 | 0.8 (1/121) | | 0.9 (2/221) | | 0 | | 0.3 (1/368) |
| 24m | 3.1 (4/129) | 2.6 (3/114) | | 0 | | 0 | | 0 |
| **15B/C** |  |  | |  | |  | |  |
| 2m | 0 | 0 | | 0 | | 1.0 (2/202) | | 0 |
| 6m | 0 | 0 | | 0 | | 2.0 (4/201) | | 0 |
| 9m | 0.7 (1/145) | 0 | | 0.4 (1/239) | | 1.0 (2/196) | | 0 |
| 12m | 0.7 (1/141) | 0 | | 0.4 (1/231) | | 2.1 (4/194) | | 2.1 (4/188) |
| 18m | 0 | 1.7 (2/121) | | 2.3 (5/221) | | 0 | | 0.5 (2/368) |
| 24m | 2.3 (3/129) | 0 | | 2.4 (5/205) | | 0.6 (1/176) | | 1.2 (2/170) |
| **34** |  |  | |  | |  | |  |
| 2m | 0 | 0 | | 0.4 (1/250) | | 0 | | 0.5 (1/197) |
| 6m | 0 | 0 | | 0.4 (1/243) | | 0.5 (1/201) | | 1.0 (2/193) |
| 9m | 0.7 (1/145) | 1.5 (2/137) | | 0 | | 1.0 (2/196) | | 2.6 (5/190) |
| 12m | 0 | 2.3 (3/133) | | 0.9 (2/231) | | 0.5 (1/194) | | 3.2 (6/188) |
| 18m | 0 | 0.8 (1/121) | | 0.9 (2/221) | | 2.1 (4/189) | | 1.9 (7/368) |
| 24m | 2.3 (3/129) | 0.9 (1/114) | | 0.5 (1/205) | | 0 | | 2.9 (5/170) |
| **Other non-vaccine serotypes** | | |  | |  | |  |  |
| 2m | 0 | 0.7 (1/149) | | 0 | | 0.5 (1/202) | | 1.0 (2/197) |
| 6m | 0 | 2.2 (3/139) | | 1.6 (4/243) | | 1.5 (3/201) | | 0 |
| 9m | 0.7 (1/145) | 0 | | 1.3 (3/239) | | 1.5 (3/196) | | 0 |
| 12m | 2.1 (3/141) | 1.5 (2/133) | | 1.7 (4/231) | | 3.1 (6/194) | | 1.6 (3/188) |
| 18m | 0.7 (1/134) | 0 | | 0.9 (2/221) | | 0.5 (1/189) | | 0.5 (2/368) |
| 24m | 2.3 (3/129) | 0.9 (1/114) | | 0.5 (1/205) | | 1.1 (2/176) | | 0.6 (1/170) |

Data are prevalence, expressed as a percentage, (n/N). * Control data sourced from Group F (2-12 months), Groups F and G combined (18 months), or Group G (24 months). Carriage determined by culture and latex agglutination/Quellung testing (2-12 months) and by DNA microarray (18-24 months). Samples that could not be serotyped are excluded. The **53 Other non-vaccine serotypes** comprise: **9 x serotype 3** (1 at 6m [B], 2 at 9m [D=2], 4 at 12m [C=3, D=1], 2 at 18m [D=1, Controls=1]), **8 x serotype 35B** (3 at 6m [C=1, D=2], 2 at 12m [D], 1 at 18m [C], 2 at 24m [D=1, Controls=1]), **6 x serotype 7C** (1 at 2m [B], 2 at 12m [B=1, Controls=1], 1 at 18m [A], 2 at 24m [A=1, B=1]), **5 x serotype 13** (1 at 2m [B], 2 at 6m [B=1, C=1], 2 at 18m [C=1, Controls=1]), **4 x serotype 19C** (1 at 2m [Controls], 3 at 12m [A=1, C=1, D=1]), **3 x serotype 6C** (1 at 9m [A], 1 at 12m [A], 1 at 24m [A]), **2 x serotypes 8** (2 at 12m [A=1, Controls=1]) and **9A** (1 at 6m [D], 1 at 9m [D]), and **1 x serotypes 15F** (12m [D]), **16F** (9m [C]), **17F** (12m [Controls]), **19** (9m [C]), **19B** (2m [Controls]), **20** (9m [C]), **23B** (12m [B]), **28F** (2m [D]), **35A** (24m [A]), **35F** (6m [C]), **37** (6m [C]), **38** (24m [C]), **42** (24m [D]), and **45** (12m [D]). PCV = pneumococcal conjugate vaccine. PCV10 = ten-valent PCV.

# **Appendix Figure S1: Pneumococcal density at A) 18 months of age, and B) 24 months of age.**

**B) 24 months**

**A) 18 months**


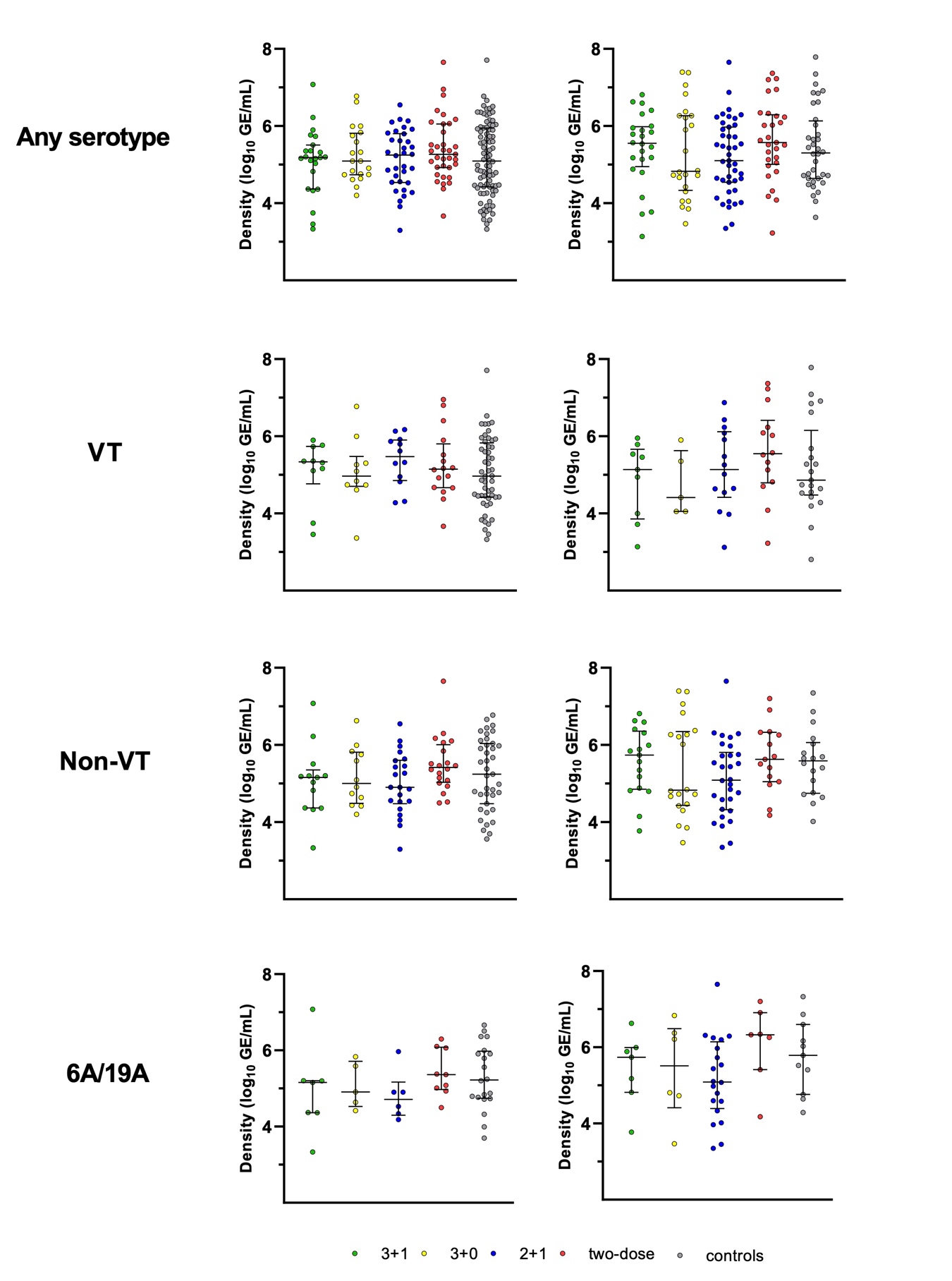


Scatter plots of density of carriage of any pneumococcal serotype, VT serotypes, non-VT serotypes, and serotypes 6A or 19A (log_10_ genome equivalents per ml), among pneumococcal carriers at 18 and 24 months of age. Circles indicate data points, and solid bars indicate the median and interquartile range. VT = vaccine type (PCV10 serotypes 1, 4, 5, 6B, 7F, 9V, 14, 18C, 19F, and 23F). Control group data come from: Groups F and G combined (18 months); or Group G (24 months).

# **Appendix Table S5: Comparative effect of a three-dose or two-dose primary series on VT carriage during the interval between the primary series and the booster doses**

|  | **VT carriage prevalence, % (95% CI)** | |  | **Prevalence ratio (95% CI)** | **p-value** |
| --- | --- | --- | --- | --- | --- |
|  | 3-dose primary series (Groups A and B combined) | 2-dose primary series (Group C) |  |  |  |
| 6 months | 2·8 (1·2-5·5) | 4·9 (2·6-8·5) |  | 0·57 (0·24-1·37) | 0·254 |
| 9 months | 5·7 (3·3-9·1) | 2·9 (1·2-5·9) |  | 1·94 (0·81-4·63) | 0·140 |

p-values based on Fisher’s Exact test (two-sided). VT = vaccine type (PCV10 serotypes 1, 4, 5, 6B, 7F, 9V, 14, 18C, 19F, and 23F).

# **Appendix Table S6: Effect of a booster-dose on VT carriage at 12, 18, and 24 months of age**

Comparison of a 3+1 and 3+0 schedule, and comparison of combined data from the 3+1 and 2+1 schedules with a 3+0 schedule

|  | **VT carriage prevalence, % (95% CI)** | | |  | **3+1 vs 3+0** | |  | **3+1/2+1 vs 3+0** | |
| --- | --- | --- | --- | --- | --- | --- | --- | --- | --- |
|  | **3+1 schedule**  (Group A) | **3+1/2+1 schedule**  (Groups A and C combined) | **3+0 schedule**  (Group B) |  | **Prevalence ratio**  **(95% CI)** | **p-value** |  | **Prevalence ratio**  **(95% CI)** | **p-value** |
| 12 months | 6·3 (2·9-11·7) | 5·9 (3·7-8·8) | 7·5 (3·7-13·4) |  | 0·84 (0·35-2·01) | 0·813 |  | 0·78 (0·38-1·61) | 0·535 |
| 18 months | 7·5 (3·6-13·3) | 6·2 (3·9-9·2) | 8·3 (4·0-14·7) |  | 0·90 (0·39-2·09) | 0·820 |  | 0·75 (0·37-1·54) | 0·409 |
| 24 months | 7·0 (3·2-12·8) | 6·9 (4·4-10·2) | 4·4 (1·4-9·9) |  | 1·59 (0·55-4·61) | 0·423 |  | 1·57 (0·61-4·03) | 0·501 |

p-values based on Fisher’s Exact test (two-sided). VT = vaccine type (PCV10 serotypes 1, 4, 5, 6B, 7F, 9V, 14, 18C, 19F, and 23F).
