# Supplementary material for: Effect of different schedules of ten-valent pneumococcal conjugate vaccine on pneumococcal carriage in Vietnamese infants: results from a randomised controlled trial
Source: Lancet Reg Health West Pac. 2022 Dec 3;32:100651. doi: 10.1016/j.lanwpc.2022.100651 (PMC9918756; doi:10.1016/j.lanwpc.2022.100651)
Supplement: Caption for supplementary material [file mmc2.docx]

Appendix

Supplementary tables and figures
